# Soft and Hard Iron Compensation for the Compasses of an Operational Towed Hydrophone Array without Sensor Motion by a Helmholtz Coil

**DOI:** 10.3390/s21238104

**Published:** 2021-12-03

**Authors:** Tommaso Lapucci, Luigi Troiano, Carlo Carobbi, Lorenzo Capineri

**Affiliations:** 1 Department of Information Engineering, University of Florence, 50139 Florence, Italy; tommaso.lapucci@stud.unifi.it (T.L.); carlo.carobbi@unifi.it (C.C.); 2 Centre for Maritime Research and Experimentation-NATO-STO, 19126 La Spezia, Italy; Luigi.Troiano@cmre.nato.int

**Keywords:** magnetic instruments, digital compass, soft and hard iron compensation, Helmholtz coil, towed hydrophone array

## Abstract

Usually, towed hydrophone arrays are instrumented with a set of compasses. Data from these sensors are utilized while beamforming the acoustic signal for target bearing estimation. However, elements of the hydrophone array mounted in the neighborhood of a compass can affect the Earth’s magnetic field detection. The effects depend upon the materials and magnetic environment present in the vicinity of the platform hosting the compass. If the disturbances are constant in time, they can be compensated for by means of a magnetic calibration procedure. This process is commonly known as soft and hard iron compensation. In this paper, a solution is presented for carrying out the magnetic calibration of a COTS (Commercial Off the Shelf) digital compass without sensor motion. This approach is particularly suited in applications where a physical rotation of the platform that hosts the sensor is unfeasible. In our case, the platform consists in an assembled and operational towed hydrophone array. A standard calibration process relies on physical rotation of the platform and thus on the use of the geomagnetic field as a reference during the compensation. As a variation on this approach, we generate an artificial reference magnetic field to simulate the impractical physical rotation. We obtain this by using a tri-axial Helmholtz coil, which enables programmability of the reference magnetic field and assures the required field uniformity. In our work, the simulated geomagnetic field is characterized in terms of its uncertainty. The analysis indicates that our method and experimental set-up represent a suitably accurate approach for the soft and hard iron compensation of the compasses equipped in the hydrophone array under test.

## 1. Introduction

The towed hydrophone array (THA) consists essentially of a line of hydrophones mounted inside a flexible hose that is towed by a submerged or surface vessel. Some of the advantages of such arrays are the large aperture at a low frequency of operation and the reduction of susceptibility to vessel noise [1]. However, it cannot be assumed that the hydrophones lie in a straight line behind the towing vessel. The correct measurement of Magnetic North is usually the only information available for signal processing on the received acoustic waves [2]. Thus, several digital compasses containing triaxial magnetometers and triaxial accelerometers are mounted in the array to provide heading information along its length.

In a digital compass, the accelerometer measures the gravitational vector and the magnetometer measures the Earth’s magnetic field vector. Measurements made by the latter can be influenced by any object mounted near the sensor that can affect the Earth’s magnetic field.

This effect depends on the system under test, but as long as the distortions are stationary in time and space, they can be taken into account through a magnetic calibration. This process is commonly known as soft and hard iron compensation. The terms hard and soft refer to the magnetic properties of the material generating the distortion, particularly to the strength of the magnetic field needed to align the magnetic domains.

It is important to note that the calibration of magnetometers with Helmholtz coils is a well known technique, adopted by sensor manufacturers to calibrate their products [3,4,5]. These manufacturers also provide software to allow hard and soft iron compensation of their sensors when mounted in the final application. However, these software routines invariably rely on the physical rotation of the entire platform in which the sensor is mounted. The current work allows the use of the sensor manufacturer’s standard magnetic calibration software but avoids the physical rotation of the platform, which, in the case of a THAs, is often greater than 50 m in length. The work therefore adopts a known method but applies it, with the aid of manufacturer’s calibration software, to the calibration of THA compasses in the laboratory environment. Hard magnetic materials have a wide hysteresis loop, so they have a high residual magnetization and cannot be easily demagnetized. Importantly, this means that they can maintain their magnetic induction regardless of the presence of an external magnetic field in the range of Earth’s field [6]. Soft magnetic materials are characterized by a narrow hysteresis loop and therefore they can be easily magnetized and demagnetized [7]. In this case, the magnitude and direction of the induced field changes according to the magnitude and direction of the external magnetic field. Consequently, the two groups have a different effect on the outputs of the magnetometers. The usual calibration procedure relies on the physical rotations of the compass and the host platform in the Earth’s magnetic field to obtain an estimation of soft and hard iron distortions superimposed on the Earth’s field. Indeed, by mapping the magnetometer outputs, the errors caused by these disturbances can be calculated with numerical techniques and removed by adjusting the digital compass outputs. The process assumes that varying the orientation of the sensor in a non-disturbed geomagnetic field, all measured values of magnetic field would ideally lay on an axis-centered sphere, whereas in the presence of soft and hard iron distortions, they lay on a shifted ellipsoid. Calibration software derives a function to fit the measured ellipsoid to the reference sphere and uses this function to create a set of magnetometer calibration parameters [8,9]. We wish to point out that the soft and hard iron compensation is a well defined problem, and manufacturers provide documents to describe how they deal with this issue in their sensors. In this regard, we found, in [10], a useful reference document. Moreover, several researchers are continuing to study the effect of the disturbances on magnetic sensors [11] and how to implement new and more accurate techniques for compass calibration [12,13,14]. However, the state of the art lacks examples and case-studies of when the hosting platform is bulky and cannot be physically rotated. This is the original contribution of the present work. In the next sections, we describe how we dealt with this problem and the formal analysis of the calibration accuracy based on the sensors and electronics is reported.

## 2. Soft and Hard Iron Compensation Simulating Towed Hydrophone Array Motion

To carry out a soft and hard iron compensation that considers all the magnetic distortions present in the THA, calibration the compasses must be carried out in its fully operational configuration; all sensors (including the hydrophone channels with their signal conditioning and digitizing electronics) must be powered on. This will ensure that any current carrying conductors in the vicinity of the sensor (producing hard iron distortion) will also be compensated. Subsequently, in line with the compass manufacturers’ procedure, users would then have to rotate the entire system in an environment with a uniform Earth magnetic field. This is impractical in the case of a THA for reasons already stated. Therefore, our approach is to generate an artificial reference magnetic field as a stimulus for the compensation. Indeed, by placing the segment of the array containing the compass inside a tri-axial Helmholtz coil (HHC) and generating a rotation in the space of a uniform magnetic field, the physical rotation may be simulated. The main design requirements for our application are:

(1) To generate a magnetic field sufficiently homogeneous inside a region. This region shall be large to contain the segment of the hydrophone array;
(2) To be able to produce a uniform magnetic field in any direction;
(3) To be reprogrammable through a PC since the field produced by the laboratory set-up depends upon the location;
(4) To generate a magnetic induction comparable with the Earth’s magnetic flux density (i.e., about 50 μT or 500 mG).

**Figure 1:**
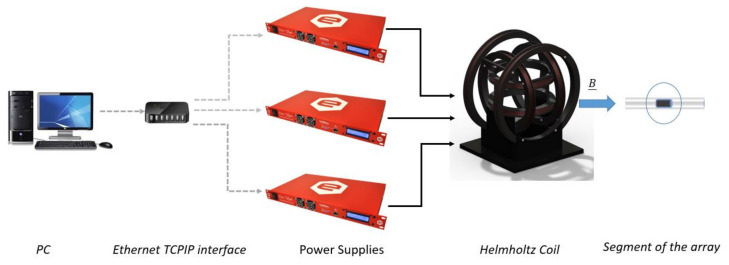
Main components and connections of the experimental set up.

To account for the first two requirements, we used a tri-axial Helmholtz Coil (Model: HHC Spin-Coil series 7-9-11-XYZ by Micro Magnetics, Inc. 617 Airport Road, Fall River, MA, USA, [15]) whose specifications report a uniform field region, with a negligible 0.4% tolerance, within a sphere with a 4.45-cm diameter that is suitable for slim THAs with a diameter of about 3 cm [16]. Moreover, bipolar power supplies are required to allow reversal of the current direction. To make the system easy to configure, three digitally controlled power supplies (Model: easy-driver 0112 by caen els) were selected to drive the coils. The power supplies are controlled via Ethernet TCP/IP protocol, using the matlab Instrument Control Toolbox. A block diagram of the main components of the calibration system is shown in Figure 1.

**Figure 2:**
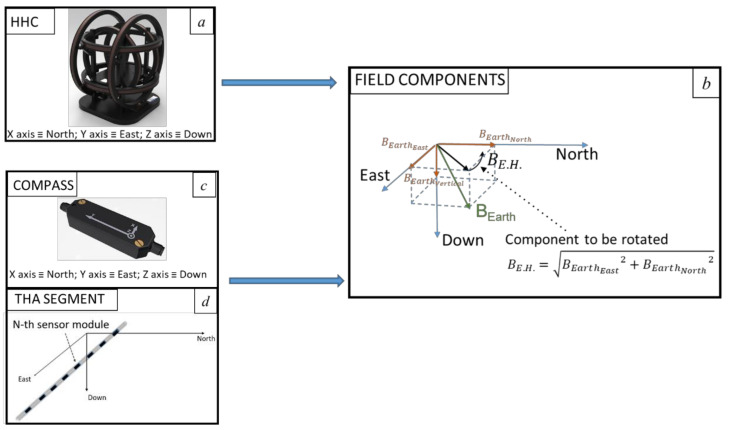
Set-up alignments procedure. (**a**) The HHC produces the field components. (**b**) Definition of the field components in a NED (East, North, Down) reference frame. (**c**,**d**) The THA segment and the compass are subject to the field components. Drawing and pictures are not to scale.

Our approach aims to simulate the rotation of the host platform and the sensor whilst continuing to use the magnetic calibration’s software provided with the compass. In this regard, it must be pointed out that a digital compass is a six-axis device that integrates a three-axis magnetometer and a three-axis accelerometer. The device incorporates an accelerometer to obtain tilt information: i.e., to detect the roll and pitch angles between the sensor’s reference frame and the local horizontal plane defined perpendicularly to the gravity vector, whereas magnetometers sense the Earth’s magnetic field to measure the heading angle, that is, the relative angle between Magnetic North, and the projection of the longitudinal axis of the sensor into the local horizontal plane [17]. Due to this computation and given that the calibration’s software uses accelerometer data, the only rotation that one can simulate is the one around the vertical axis (aligned with gravity) as the outputs of the accelerometers are expected to remain constant in any case. Therefore, aligning our set-up to a North-East-Down (NED) frame, the HHC must be able to perform a rotation of the horizontal components of the Earth’s field whilst maintaining the vertical component constant (see Figure 2). This is not considered a limitation for THAs since when correctly trimmed (i.e., neutrally buoyant), they are always horizontal during operational use.

## 3. Experimental Procedure

**Figure 3:**
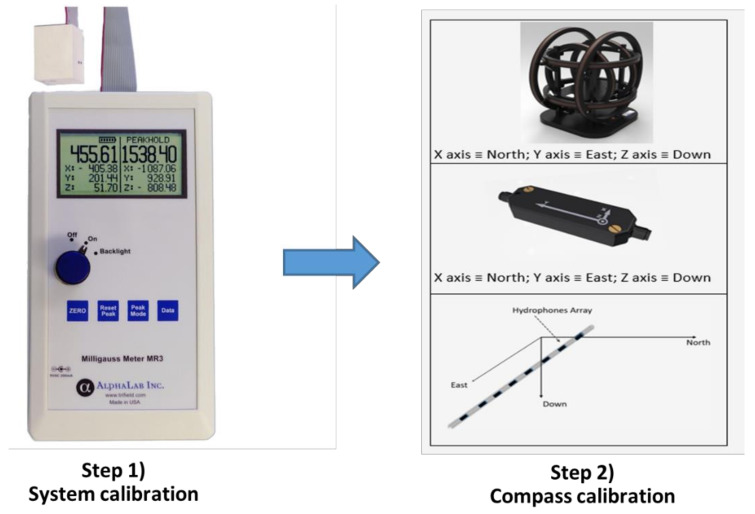
The two-step procedure needed to calibrate our set-up. Step (**1**) System calibration: the milligauss MR3 m is placed inside the tri-axial Helmholtz coil (HHC). Step (**2**) Compass calibration: the segment of the towed hydrophone array (THA) where the compass is mounted is placed inside the HHC.

With the aim of generating an artificial field which simulates the rotation of the THA around the vertical axis, a two-step procedure was developed (Figure 3). Firstly, there is the “System calibration” step that it is carried out with an auxiliary 3-Axis Magnetoresistive Milligauss Meter (model MR3 by AlphaLab) positioned at the center of the HHC with the hypothesis of no axis misalignment. This meter has superior specifications to those of the magnetometers used in the THAs digital compasses. Secondly, there is the “Compass calibration” step with the THA segment including the compass inside the HHC.

The first step is performed through a test rotation and magnetic field measurements taken with the MR3 m. The aim is to remove possible systematic error sources of our experimental system, to measure the local background field and to verify the effect of the background field. Indeed, the background field should not be affected by the change in field generated by the HHC. The implication is that the effect of the background field should be limited to the hard iron type, since a soft iron would provide different perturbations in the first and second steps. Thus, background field compensation as well as care in positioning the coils in a suitable environment, where there are no magnetically soft materials surrounding the HHC, are the main tasks of this activity.

During the test rotation, the system is used to generate the desired calibration values of magnetic field. We define the desired components (*B_Earth_East_*, *B_Earth_North_, B_Earth_Vertical_*) according to our location, through the model provided by the International Geomagnetic Reference Field (IGRF). Driving X (North) and Y (East) coils with harmonic time-dependent currents produces the rotation of the vector around the vertical.

(1) $$B_{x_{d}}\left(t_{i}\right)=\;B_{E.H.}\times \left(cos\omega t_{i}\right)$$

(2) $$B_{y_{d}}\left(t_{i}\right)=\;B_{E.H.}\times \left(sin\omega t_{i}\right)$$

(3) $$B_{z_{d}}\left(t_{i}\right)=\;B_{Earth_{Vertical}}$$

where *B_E.H._* is defined in Figure 2 and ω is the angular frequency of the artificial rotation of the magnetic field vector. In practice, the sinusoidal waveform is discretized. The number of steps and step intervals is chosen to allow the system to settle before taking a measurement with the MR3 gauss meter. It was determined that sending commands to power supplies with an interval of 0.5 s and a period of 60 s was a suitable trade-off.

A set of parameters are subsequently extracted from the measurements $B_{x,y,z_{MR3_{i}}}$ taken with the Milligauss meter during the test rotation. These parameters are required to overcome the background field, check that there is no soft iron effect in the background and calibrate the system. The method is used to measure the eccentricity through $SF_{x}$ and $SF_{y}$ and the offset of the test rotation:

(4) $$SF_{x}=\frac{2B_{E.H.}}{max\left(B_{x_{MR3_{i}}}\right)-min\left(B_{x_{MR3_{i}}}\right)}$$

(5) $$SF_{y}=\frac{2B_{E.H.}}{max\left(B_{y_{MR3_{i}}}\right)-min\left(B_{y_{MR3_{i}}}\right)}$$

(6) $$B_{Off_{x}}=\frac{\sum_{i=1}^{N}B_{x_{MR3_{i}}}}{N}$$

(7) $$B_{Off_{y}}=\frac{\sum_{i=1}^{N}B_{y_{MR3_{i}}}}{N}$$

(8) $$B_{Off_{z}}=\frac{\sum_{i=1}^{N}B_{z_{MR3_{i}}}}{N}-B_{z_{d}}$$

with the aid of the previous steps, we can now perform the rotation of the field, which simulates a physical rotation in a uniform “Earth’s Field”. To make this, we require from the coils the field components expressed by the following equations:

(9) $$B_{x_{c}}\left(t_{i}\right)=\;SF_{x}\times \left(B_{E.H.}\times \left(cos\;cos\;\omega t_{i}\;\right)-B_{Off_{y}}\right)$$

(10) $$B_{y_{c}}\left(t_{i}\right)=\;SF_{y}\times \left(B_{E.H.}\times \left(sin\;sin\;\omega t_{i}\;\right)-B_{Off_{y}}\right)$$

(11) $$B_{z_{c}}\left(t_{i}\right)=\;B_{Earth_{Vertical}}-B_{Off_{z}}$$

During both steps, the input currents *I* that are requested to the power supplies are computed through:

(12) $$I_{x,y,z}\left(t_{i}\right)=\frac{B_{x,y,z}\left(t_{i}\right)}{k_{x,y,z}}$$

where *k* is the coil-constant provided by the HHC manufacturer [15]. The driving current during the compass calibration step depends upon several factors including the background field, which cannot be foreseen a priori and changes according to the location. In our test, the sinusoidal driving current has a peak-to-peak value of tens of mA and the 16-bit resolution of the power supplies allows to generation of the required 120 samples (60 s period with a command rate of 0.5 s) in this current range.

The accuracy limit of the procedure is given by the auxiliary magnetometer that has been used as a reference. The influence of this parameter is discussed in the next section.

## 4. Discussion on the Artificially Generated Magnetic Field

**Figure 4:**
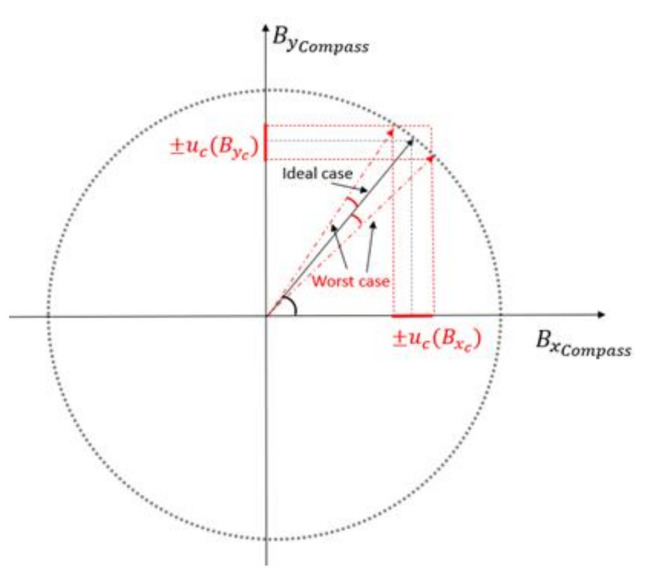
The worst-case scenario is reached when $B_{x_{compass}}$ and $B_{y_{compass}}$ have values equal to the nominal plus (minus) $u_{c}\left(B_{x_{c}}\right)$ and minus (plus) $u_{c}\left(B_{y_{c}}\right)$ respectively.

**Figure 5:**
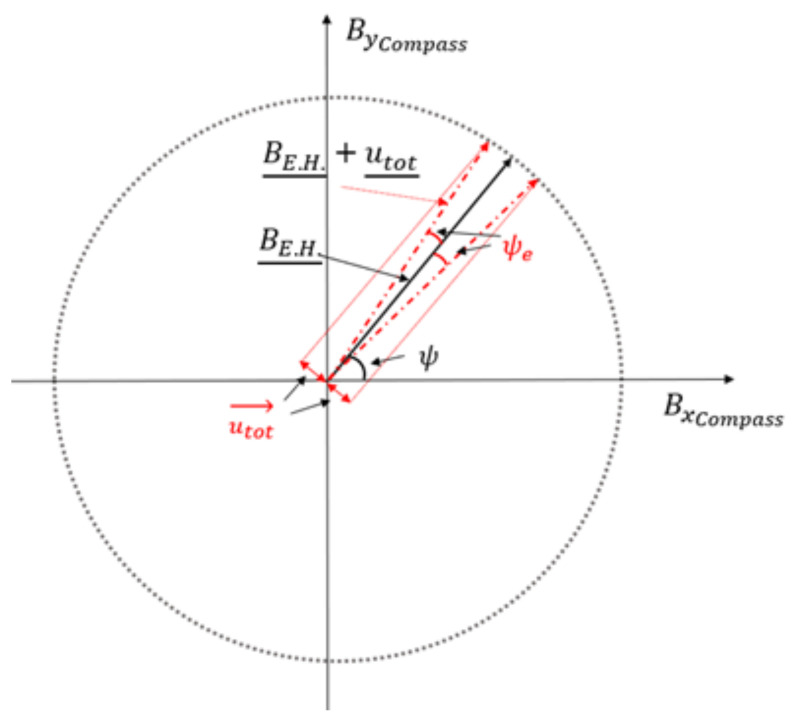
The worst-case scenario provides a maximum heading error that can be computed through the vectors shown here.

As a variation on the standard calibration, which relies on a real geomagnetic field, we expose the sensor to an artificial field. Although the artificial field has a small degree of non-uniformity, in the soft and hard iron compensation, the important requirement is that the modulus of the stimulus used as a reference be constant during the calibration; the actual value is less important. The reference vectors we provide for the compass calibration step are affected by an uncertainty deriving from the measurements taken with the MR3 m for the system calibration. Such uncertainty is tolerable if small enough to allow the assessment of compliance of the compass to be compensated within its specifications. In this regard, it is worth noting that the manufacturer’s procedure for soft and hard iron compensation is intended to take place directly in the Earth’s magnetic field. Therefore, the compass datasheet does not report specifications required for the field used as a stimulus during compensation. In our case, we will evaluate the uncertainty of the components $B_{x_{c}},B_{y_{c}}$, as acceptable, providing it induces a heading error lower than the accuracy of the sensor to be calibrated. As reported in Figure 4, the worst-case scenario is when $B_{x_{c}},B_{y_{c}}$ are subject to the maximum value of the uncertainties with opposite signs. This is equivalent to summing to the desired vector, an orthogonal vector, with a magnitude equal to the vector addition of the uncertainties (Figure 5):

(13) $$\left|\underline{u_{tot}}\right|=\sqrt{u_{B_{x_{c}}}^{2}+u_{B_{y_{c}}}^{2}}$$

Then, the assessment of the uncertainties on $B_{x_{c}},B_{y_{c}}$ allows evaluation of the maximum heading error $\psi_{e}$ of our system through the scalar product between the two resulting vectors $\underline{B_{E.H.}}$ and $\underline{B_{E.H.}}+\underline{u_{tot}}$:

(14) $$cos\psi_{e}=\frac{\underline{B_{E.H.}}\cdot \left(\underline{B_{E.H.}}+\underline{u_{tot}}\right)}{\left|\left(\underline{B_{E.H.}}+\underline{u_{tot}}\right)\right|\left|\underline{B_{E.H.}}\right|}$$

## 5. Uncertainty Analysis

This section reports the overall result of our system in producing the reference vectors for the compensation on soft and hard iron distortions. As stated previously, this will be reported in terms of standard uncertainty on the artificial geomagnetic field components set to perform the final rotation. Formally, this has to be expressed in terms of analytical relations (Equations (9) and (10)) between the measurands, $B_{x_{c}}$ and $B_{y_{c}}$, and the input quantities on which the measurands depend, $B_{x_{MR3_{i}}}$ and $B_{y_{MR3_{i}}}$. In our case, no substantial variation in the output of our measurements is evident, other than slow drifts in the environmental conditions which are considered small on the time scale of our calibration procedure. Hence, we make a Type B uncertainty evaluation and take the reading as conventional true value [18]. The user manual of the AlphaLab MR3 magnetometer reports an 𝐴𝑐𝑐𝑢𝑟𝑎𝑐𝑦 ($B_{MR3_{i}}$) = ±0.5% × 𝑅𝑒𝑎𝑑𝑖𝑛𝑔𝑠. Assuming a uniform probability density function, the type B standard uncertainty for a recorded value $B_{MR3_{i}}$ is

(15) $$u_{b}(B_{MR3}{}_{i})=0.5\%\times \frac{B_{MR3}{}_{i}}{\sqrt{3}}$$

We evaluate the parameters in Equations (6)–(9) through correlated input quantities without knowing the correlation coefficient, so we compute the worst-case combined uncertainty. This is formally expressed by

(16) $$u_{c}=\overset{N}{\underset{i=1}{\sum }}\left|\frac{\partial g\left(n_{1_{0}},n_{2_{0}},\ldots ,n_{m_{0}}\right)}{\partial n_{i}}\right|u_{n_{i}}$$

where $g\left(n_{i}\right)$ is the actual relation between the measurements and the measurand and $n_{1_{0}},n_{2_{0}},\ldots ,n_{m_{0}}$ are the conventional true values of the N measurements [18].

Thus, the standard uncertainty on the components required for Equation (13) is computed as

(17) $$u_{c}\left(B_{x_{c}}\right)=\left|B_{E.H.}\times \left(cos\;cos\;𝜔t\right)-B_{Off}\right|\times u_{SF}+SF\times u_{B_{Off}}$$

(18) $$u_{c}\left(B_{y_{c}}\right)=\left|B_{E.H.}\times \left(sin\;sin\;𝜔t\right)-B_{Off}\right|\times u_{SF}+SF\times u_{B_{Off}}$$

These equations have the following maximum value that will be used to evaluate our system in the worst-case scenario:

(19) $$u_{c}\left(B_{c}\right)\leq \left|B_{E.H.}-B_{Off}\right|\times u_{SF}+SF\times u_{B_{Off}}$$

After performing several tests in our site, we obtained a maximum value of 2 mG for Equation (19). Using Equation (14), we found a worst-case scenario of 0.6° of heading error that is lower than 1°, that is, the typical heading accuracy for the COTS compasses that are mounted on the THA under test.

## 6. Conclusions

This work led to the definition of a procedure and an experimental set-up to perform the soft and hard iron compensation on the compasses mounted in a towed hydrophone array. The novelty of this work lies in a calibration procedure that does not rely on an unfeasible sensor motion; it permits keeping the array assembled and operational whilst calibrating the compasses. This has the benefit of compensating for the full range of interference sources experienced by the THA in during normal operation, factors which cannot be considered if the compass is calibrated prior to mounting in the THA. Sea trials of THAs containing compasses calibrated using this technique will, in the future, be evaluated in the field.

## References

[1] Barbagelata A., Guerrini P., Troiano L. (2008). Thirty Years of Towed Arrays at NURC. Oceanography.

[2] Felisberto P., Jesus S.M. (1996). Towed array beamforming during ship’s maneuvering. IEE Radar Sonar Navig..

[3] Calibration of Compasses Using Helmholtz Coil Systems. Application Note. https://www.bartington.com/wp-content/uploads/pdfs/application_notes/AN0051.pdf.

[4] Zikmund A., Janosek M., Ulvr M., Kupec J. (2015). Precise Calibration Method for Triaxial Magnetometers Not Requiring Earth’s Field Compensation. IEEE Trans. Instrum. Meas..

[5] https://patents.google.com/patent/WO2018006020A1/en.

[6] Liu Z., Zhang Q., Pan M., Shan Q., Geng Y., Guan F., Chen D., Tian W. (2017). Distortion Magnetic Field Compensation of Geomagnetic Vector Measurement System Using a 3-D Helmholtz Coil. IEEE Geosci. Remote Sens. Lett..

[7] Inoue A., Kong F. (2022). Soft Magnetic Materials. Encycl. Smart Mater..

[8] Xsens Magnetic Calibration Manual. Document MT0202P. https://www.xsens.com/hubfs/Downloads/Manuals/MT_Magnetic_Calibration_Manual.pdf.

[9] Garton M., Wutka A., Leuzinger A. (2009). Local Magnetic Distortion Effects on 3-Axis Compassing. PNI Sens. Corp. Tech. Rep..

[10] Calibrating an eCompass in the Presence of Hard-and Soft-Iron Interference. Application Note Document Number: AN4246 Rev. 4.0, 11/2015. https://www.nxp.com/docs/en/application-note/AN4246.pdf.

[11] Fan B., Li Q., Tao L. (2018). How Magnetic Disturbance Influences the Attitude and Heading in Magnetic and Inertial Sensor-Based Orientation Estimation. Sensors.

[12] Papafotis K., Nikitas D., Sotiriadis P.P. (2021). Magnetic Field Sensors’ Calibration: Algorithms’ Overview and Comparison. Sensors.

[13] Fang J., Sun H., Cao J., Zhang X., Tao Y. (2011). A novel calibration method of magnetic compass based on ellipsoid fitting. IEEE Trans. Instrum. Meas..

[14] Liu Y.X., Li X.S., Zhang X.J., Feng Y.B. (2014). Novel Calibration Algorithm for a Three-Axis Strapdown Magnetometer. Sensors.

[15] MicroMagnetics SpinCoil 7-9-11-XYZ: Three-Axis Helmholtz Coils User Manual Rev.3. http://www.micromagnetics.com/products_hhccontrol.html.

[16] Maguer A., Dymond R., Mazzi M., Biagini S. (2008). SLITA: A new slim towed array for AUV applications. J. Acoust. Soc. Am..

[17] Caruso M.J. Applications of magnetic sensors for low cost compass systems. Proceedings of the IEEE 2000 Position Location and Navigation Symposium.

[18] Joint Committee for Guides in Metrology (2008). Evaluation of measurement data—Guide to the expression of uncertainty in measurement. JCGM.

